# Defining the student perspective on radiation oncology—an analysis of factors influencing medical students’ decisions for specialized training

**DOI:** 10.1007/s00066-025-02396-x

**Published:** 2025-04-10

**Authors:** Stefan Gravemeyer, Dogus Darici, David Rene Steike, Martina Schmitz, Hans Th. Eich, Michael Oertel

**Affiliations:** 1 Department of Radiotherapy and Oncology, University hospital of Munster, Munster, Germany; 2 Institute for Anatomy and Molecular Neurobiology, University of Munster, Munster, Germany

**Keywords:** Radiotherapy, Medical school, Career, Students’ opinion

## Abstract

**Purpose:**

The new Medical Licensing Regulations 2025 in Germany demand a longitudinal and interdisciplinary oncological curriculum for the future of medical education. Small disciplines like radiation oncology (RO) tend to be underrepresented in the general curriculum, which complicates attracting new residents and doctoral candidates to the field. To bridge this gap, our university successfully implemented a multidisciplinary training for preclinical semesters combining anatomical and RO knowledge. The following study addresses students’ perceptions of RO and learning success in the setting of a mandatory preclinical course.

**Methods:**

A quantitative single-center cross-sectional study with 106 students was conducted via online questionnaire before and after a 1-week semester course in anatomy and RO. The analysis was conceptualized using descriptive statistical methods and the expectancy–value model according to Eccles and Wigfield.

**Results:**

Overall, 106 (73 female, 33 male) students with a mean age of 21.8 years took part in the first survey. Advanced courses during finals and gender had no effect on interest in RO. However, it could be shown that the understanding of RO (*p* < 0.0001), knowledge about patients who need to be referred to RO (*p* < 0.0001), and the interest in specialty training in RO (*p* < 0.0001) significantly increased during the course*. *The students’ perceptions of specialty training in RO remained stable before and after the course.

**Conclusion:**

This is the first study on the influence factors for students’ decisions to pursue a specialized training in RO. Students’ expectations regarding a good specialty training are well represented in RO, and the implementation of preclinical courses significantly increases the knowledge about RO and the level of interest regarding a specialty training in RO.

## Introduction

After graduation from medical school, former students decide in which medical specialty they would like to start their specialized training. As of today (September 2024), there are 51 medical specialities available in 34 fields to choose from in Germany [1]. Of the 421,303 practicing physicians at the end of 2022, only 1564 (around 0.4%) worked in the field of radiation oncology (RO) [2]. However, around half of all cancer patients require radio-oncological treatment during the course of their disease. Together with technical and conceptual innovations within the field, this creates an increased demand for qualified radiation oncologists [3]. Due to this trend, national radiation oncology societies such as the German Society of Radiation Oncology (DEGRO) or the Canadian Association of Radiation Oncology (CARO) have initiated taskforces to position themselves for the future of cancer care [4–6]. In contrast, this trend is not reflected in the curricula of medical studies [7]. According to medical licensure laws in Germany, training in RO plays only a small part in the curricular teaching represented in cross section 11, “Imaging techniques, radiation oncology, radiation protection” [8]. For this reason, RO has a challenging position among students—both in terms of distinguishing itself from the fields of radiology and nuclear medicine as well as in terms of its perception as a distinct clinical oncological discipline. In most cases, radiation oncology training is offered only in the second half of medical education during clinical semesters [9]. Predominantly, teaching takes place in the form of lectures or bedside teaching [9, 10]. More and more faculties are expanding their teaching offers in RO by digital and non-digital formats for medical students [11–14] to realize the aim of a longitudinal and interdisciplinary oncological curriculum according to the standards of the new Medical Licensing Regulations 2025, which stipulate an integration of clinical subjects into preclinical training [7, 8, 15, 16]. Following this trend, there has been an increase in generally voluntary programs that also address training in RO in preclinical studies [17–19], although it has been shown that the closure of knowledge gaps is especially possible with mandatory teaching concepts [20]. In 2015, the University of Munster expanded a successful pilot project [21] to address both anatomical and RO knowledge as a multidisciplinary training for preclinical semesters, e.g., correlating knowledge about mediastinal anatomy with thoracic computer tomography scans and with clinical knowledge, e.g., about superior vena cava syndrome. This evolved into a successful concept within the mandatory preclinical curriculum [22].

Students decide which specialty to pursue on a multifactorial basis that includes the perception of specialty characteristics, which are influenced by the curricular teaching as well as their personal career ideas [23–30]. With targeted and early support, it is thus possible to assist motivated students in their decision to pursue a career in the field of RO [17, 31]. Questions arise as to whether there are specific groups, like students with advanced courses in natural sciences in general or, e.g., in physics, who already exhibit an inclination towards our field in preclinical semesters and what perceptions these and other students have of our field. Similarly, the question arises of whether subjective learning success, which is the main focus of implementing our field in the preclinical domain, is measurable.

The current study uses the expectancy–value model to investigate factors influencing medical students’ inclinations towards a career in RO. The expectancy–value model, developed by Eccles and Wigfield [32], is a motivational theory that explains behavior (i.e., the intention or decision to be a radiation oncologist) as a product of expectation (i.e., belief of succeeding in this area) and value (i.e., importance, interest, or utility in this area). Thus, the interplay between expectancy and value determines the motivation behind choosing a particular specialty. For example, a student with high confidence in their ability to excel in RO (high expectancy) and who also finds the field important, interesting, and beneficial (high value) is more likely to pursue a career in this area. This model can also aid in identifying potential gaps between students’ expectancies and values as well as the barriers that may discourage preclinical students from considering this specialty in the future.

The following analysis addresses these questions in the setting of a mandatory preclinical course after the third semester of our curriculum. A pre- and post-course evaluation and assessment are undertaken to illustrate the students’ perspectives of RO, followed by a comprehensive discussion of the existing literature.

## Methods

An anonymous online questionnaire was drafted to conduct the student survey before and after a 1-week semester course in anatomy and RO. The analysis was conceptualized using descriptive statistical methods and the expectancy–value model according to Eccles and Wigfield [32].

### Study variables

#### Sociodemographic variables

With the goal of identifying student characteristics that could be associated with the decision to pursue a career in RO, we measured the following covariates of the study group: What is your age? What is your sex (female, male, diverse)? Have you completed vocational training (yes/no)? Did you visit a natural science advanced course during your finals in secondary school (yes/no)? Did you visit an advanced course in physics during your finals in secondary school (yes/no)?

#### Intention to pursue a career in radiation oncology

One item was generated that captured the intention of preclinical medical students to pursue a career in RO on a Likert scale (1 = very unlikely to 5 = very likely): I could imagine completing my medical specialty training in radiation oncology.

#### Expectancies of being a radiation oncologist

According to the expectancy–value model, students were questioned about their expectations of becoming a radiation oncologist. These questions were designed to explore various aspects of professional work, including financial stability, career prospects, recognition, research opportunities, workload, administrative tasks, and the ability to work independently. Responses to the following questions were provided using a Likert scale from 1 = very low to 5 = very high: How would you assess the prospect of financial stability as a radiation oncologist? How would you rate the prospects for professional success in RO? How highly would you rate the recognition of radiation oncologists within the medical community? How would you rate the research opportunities in RO? How would you rate the workload in RO? How would you rate the frequency of administrative tasks (e.g., documentation, etc.) in RO? How would you rate the opportunity for independent work in RO?

#### Values in choosing a medical specialty

Next, the same areas were examined for their value to the students (Likert scale ranging from 1 = very low to 5 = very high): How important is financial stability to you when choosing a medical specialty? How important is professional success to you when choosing a medical specialty? How important is professional recognition to you when choosing a medical specialty? How important are research opportunities to you when choosing a medical specialty? How important is the question of workload to you when choosing a medical specialty? How important is the frequency of administrative tasks to you when choosing a medical specialty? How important is independent work to you when choosing a medical specialty?

#### Self-assessed knowledge in RO

In a last step, the students were questioned about their assessments of their knowledge of the profession of a radiation oncologist. The answers to the following questions were provided using a Likert scale from 1 = very low to 5 = very high: I have an understanding of what is included in the professional profile of a radiation oncologist. I have an idea of which patients I would need to refer to RO.

### Analysis

The software SPSS v. 29.0.0.0 (IBM Corp., Armonk, NY, USA) and GraphPad Prism v. 10.2.3 (GraphPad Software Inc., Boston, MA, USA) were used to analyze the data. All statistical tests were conducted two sided at a significance level of alpha = 0.05. For nonnormally distributed data, the Wilcoxon test was used. A Friedman test for multiple testing was conducted to assess pre–post differences in fully completed surveys. Pearson’s correlation was calculated to assess the relationship between the level of interest in specialty training in RO and gender and an advanced science course or advanced course in physics during finals in secondary school. Except for the analysis of pre–post differences referring to the expectancy–value model due to use of Friedman’s test, all collected data were included in the investigation, regardless of whether the survey was fully completed or not.

## Results

**Fig. 1:**
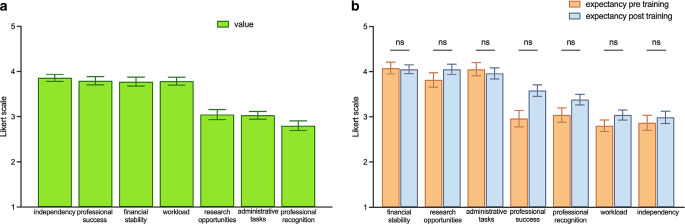
Answers to the questions according to the expectancy–value model about specialty training in radiation oncology (RO). Students’ answers about their expectations of further specialty training (**a**) and their expectations about the specialty training in RO before and after training (**b**). Data points are mean ± standard error of the mean, with significance for *p* < 0.05

Overall, 106 of 147 students (72.1%) participating in the course took part in the first survey and 83% completed every question of the survey. Of the initial group, 92 (86.7%) also took part in the second survey and 94% completed the whole survey. Regarding gender, 68.9% of participants identified as female whereas 31.1% identified as a male person. None of the students identified as diverse. Mean age was 21.8 years (18 to 33). The majority of students had attended an advanced science course during their school years (68.9%), with 10.4% of students having taken an advanced physics course. Before the course started, all participants were asked about their expectations of further specialty training. Financial stability, workload, professional success, and independency played major roles in the choice of specialty training, whereas appreciation, research opportunities, and the load of administrative tasks played minor roles (Fig. 1a).

**Fig. 2:**
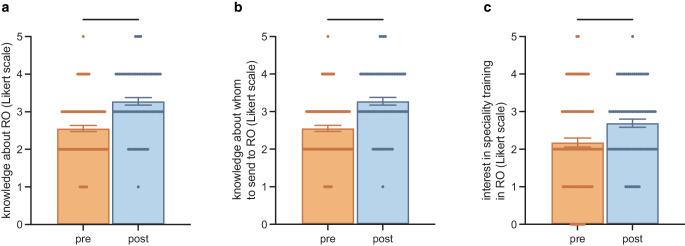
Answers to the questions about knowledge of radiation oncology (*RO*) in general (**a**), about whom to send to RO (**b**), and interest in specialty training in RO (**c**). Data points are mean ± standard error of the mean, with significance for *p* < 0.05

The students were asked about their expectations of the specialty training in RO before and after training (Fig. 1b). Financial stability and the frequency of administrative tasks were valued relatively highly in the field of RO and no difference could be seen before or after training (financial stability mean [M] = 4.2 ± 1.0 before and M = 4.1 ± 0.8 after training, *p* > 0.99; administrative tasks: M = 4.2 ± 1.2 before and M = 4.0 ± 1.1 after training, *p* > 0.99). We anticipate that the general trend toward increased documentation requirements had an impact on that question. Fortunately, the research opportunities were also valued relatively highly before (M = 3.9 ± 1.2) and after training (M = 4.1 ± 1.0), and the value of the expectation of professional success significantly increased (*p* = 0.0015), which was no longer the case when using Friedman’s test for multiple testing (*p* = 0.19; Fig. 1b). The students were asked about their understanding of what is included in the professional profile of a radiation oncologist. We could confirm that the course could significantly increase the understanding of RO (*p* < 0.0001), and participating students felt significantly safer in their knowledge regarding which patients need to be referred to RO after the course (*p* < 0.0001; Fig. 2a, b).

Following this, we could also confirm a significantly higher level of interest in specialty training in RO after the course (*p* < 0.0001; Fig. 2c). Students who attended an advanced science course during finals in secondary school did not show significantly more interest in RO before or after training (*p* = 0.436 and *p* = 0.512). Similar results were seen for students attending an advanced course in physics during finals in secondary school (*p* = 0.157 and *p* = 0.347). In addition, there was also no significant difference between students who identified male or female (*p* = 0.585 and *p* = 0.270; Table 1).

**Table 1:** Answers to the question on whether students could imagine completing their medical specialty training in radiation oncology before and after training did not differ between students identifying as male and female. Neither an advanced scientific course during finals nor an advanced course in physics had a significant impact on the results

	Before training	After training
Gender (female vs. male)	M = 2.2 ± 1.1 vs. M = 2.1 ± 1.5	M = 2.1 ± 1.4 vs. M = 1.8 ± 1.6
	*p* = 0.585	*p* = 0.270
Advanced scientific course (yes vs. no)	M = 2.3 ± 1.2 vs. M = 2.0 ± 1.3	M = 2.0 ± 1.5 vs. M = 1.9 ± 1.4
	*p* = 0.436	*p* = 0.512
Advanced course in physics (yes vs. no)	M = 2.4 ± 1.5 vs. M = 2.1 ± 1.2	M = 2.2 ± 1.5 vs. M = 2.0 ± 1.4
	*p* = 0.157	*p* = 0.347

## Discussion

The hereby presented survey is the first to monitor factors influencing students’ decisions to pursue a specialized training in RO. It demonstrates that students’ expectations regarding a good specialty training are well represented in RO and that implementation of preclinical courses can significantly increase the level of interest in the field of RO. Moreover, it is shown that with our reduced group of students who took part in the survey, we could not identify a specific group which is more interested in RO than others. In particular, there was no difference in terms of gender or in terms of advanced courses during finals. Cross-sectional surveys represent the standard tool to address the question of future career choices of medical students [33, 34]. Querido et al. defined the methodological quality of cross-sectional career choice studies with specific criteria (study purpose, study design, response rate, kind of survey, number of investigated factors, career or profession, statistical analysis) [23]. According to our evaluation, we could reach high quality in all of the named criteria when we compared our study design with highly ranked comparative studies [26, 35], except regarding the limiting factor that this was only a single-center survey. The surveyed study population was comparable to other cross-sectional surveys in Germany regarding the analyzed criteria of sex and age of preclinical medical students [24, 36]. In accordance with the literature and independently of gender or age, financial stability, workload, professional success, and independency played major roles in the choice of specialty training [23, 35]. Financial stability was valued as relatively high in the field of RO before and after training, and the value of professional success significantly increased (*p* = 0.0015) after the course, which was no longer the case when using Friedman’s test for multiple testing (*p* = 0.19). In general, these results have to be taken with a pinch of salt, because participants tend to please the investigator, but in other interventional surveys after a preclinical or clinical rotation, no positive or even negative trend could be seen [29, 37]. We anticipate that the general trend toward increased documentation requirements has an impact on the supposition that specialty training in RO is related to a high workload of administrative tasks, although in general, RO is seen as a role model for digitalization and fewer administrative tasks in modern medicine [38]. Fortunately, the research opportunities were also valued relatively highly. Brouwer et al. could confirm that with increasing progression of education, the local research opportunities increase in relevance for the choice of specialty training and residency [39].

Furthermore, we could show a higher level of interest in specialty training in RO after the course. Students also felt safer in their knowledge about the profession and in their understanding of who to send to a radiation oncologist. Similar and more objective results could also be shown with other mandatory trainings [11, 20].

Fortunately, there was no significant difference between students who identified as male or female, in contrast to most other specialties [24, 30]. To the best of our knowledge, this is the first analysis to analyze the correlation between advanced courses during finals in secondary school and specialty choice. Against our expectations, students who attended an advanced science course or even an advanced course in physics during finals in secondary school did not have an enhanced interest in RO. This is an interesting fact which needs further investigation. On the one hand, exaggerated expectations concerning knowledge in physics is a common misconception preventing students from training in RO, on the other, it seems surprising that students with an affinity to physics do not have more interest in RO [40]. A reason for the fear of physics in RO specialty training may result from the relatively large part that medical physics plays in clinical training in RO according to the actual National Competence-Based Learning Objectives Catalogue for Medicine 2.0 (NKLM) [8, 9, 13]. Due to this fact, it would also be interesting to evaluate such data during clinical training. Moreover, there is need for longitudinal studies to see if the shown short-term effects can translate into actual career choices.

Overall, RO faces a lack of future radiation oncologists concerning its expected relevance in cancer care in the upcoming years. These data provide insights into students’ views on RO and illustrate the difficulty in identifying a defined group during preclinical semesters which is likely to pursue a career in the field. Future tasks will be to integrate RO into a longitudinal and interdisciplinary oncological curriculum beginning in the first semesters, in order to generate conceptual understanding independent from the future choice of a specialty.
